# Cross-Sectional Study of Malnutrition and Associated Factors among School Aged Children in Rural and Urban Settings of Fogera and Libo Kemkem Districts, Ethiopia

**DOI:** 10.1371/journal.pone.0105880

**Published:** 2014-09-29

**Authors:** Zaida Herrador, Luis Sordo, Endalamaw Gadisa, Javier Moreno, Javier Nieto, Agustín Benito, Abraham Aseffa, Carmen Cañavate, Estefania Custodio

**Affiliations:** 1 National Centre of Tropical Medicine, Instituto de Salud Carlos III (ISCIII), Madrid, Spain; 2 Tropical Diseases Research Network (RICET in Spanish), Madrid, Spain; 3 National Centre of Epidemiology, ISCIII, Madrid, Spain; 4 Department of Preventive Medicine and Public Health, Faculty of Medicine, Complutense University, Madrid, Spain; 5 Network Biomedical Research Centers, Epidemiology and Public Health (CIBERESP in Spanish), Madrid, Spain; 6 Armauer Hansen Research Institute, Addis Ababa, Ethiopia; 7 National Centre of Microbiology, ISCIII, Madrid, Spain; University of Cape Town, South Africa

## Abstract

**Introduction:**

Little information is available on malnutrition-related factors among school-aged children ≥5 years in Ethiopia. This study describes the prevalence of stunting and thinness and their related factors in Libo Kemkem and Fogera, Amhara Regional State and assesses differences between urban and rural areas.

**Methods:**

In this cross-sectional study, anthropometrics and individual and household characteristics data were collected from 886 children. Height-for-age z-score for stunting and body-mass-index-for-age z-score for thinness were computed. Dietary data were collected through a 24-hour recall. Bivariate and backward stepwise multivariable statistical methods were employed to assess malnutrition-associated factors in rural and urban communities.

**Results:**

The prevalence of stunting among school-aged children was 42.7% in rural areas and 29.2% in urban areas, while the corresponding figures for thinness were 21.6% and 20.8%. Age differences were significant in both strata. In the rural setting, fever in the previous 2 weeks (OR: 1.62; 95% CI: 1.23–2.32), consumption of food from animal sources (OR: 0.51; 95% CI: 0.29–0.91) and consumption of the family's own cattle products (OR: 0.50; 95% CI: 0.27–0.93), among others factors were significantly associated with stunting, while in the urban setting, only age (OR: 4.62; 95% CI: 2.09–10.21) and years of schooling of the person in charge of food preparation were significant (OR: 0.88; 95% CI: 0.79–0.97). Thinness was statistically associated with number of children living in the house (OR: 1.28; 95% CI: 1.03–1.60) and family rice cultivation (OR: 0.64; 95% CI: 0.41–0.99) in the rural setting, and with consumption of food from animal sources (OR: 0.26; 95% CI: 0.10–0.67) and literacy of head of household (OR: 0.24; 95% CI: 0.09–0.65) in the urban setting.

**Conclusion:**

The prevalence of stunting was significantly higher in rural areas, whereas no significant differences were observed for thinness. Various factors were associated with one or both types of malnutrition, and varied by type of setting. To effectively tackle malnutrition, nutritional programs should be oriented to local needs.

## Introduction

Adequate nutrition is essential during childhood to ensure healthy growth, proper organ formation and function, a strong immune system, and neurological and cognitive development [1]. Nutritional status has a major impact on children's survival mainly due to the synergistic relationships between malnutrition and diseases [2], [3]. In Eastern and Southern Africa, malnutrition is a major underlying cause of the persistently high child mortality, contributing to more than a third of all deaths among children under age 5 [4].

The two main anthropometric indicators used to define malnutrition– stunting, and wasting or thinness– represent different histories of nutritional insult to the child. Linear growth retardation (chronic malnutrition or stunting) is frequently associated with repeated exposure to adverse economic conditions, poor sanitation, and the interactive effects of poor nutrient intakes and infection. Low weight-for-height or low body mass index (BMI) for age (acute malnutrition, wasting or thinness) is generally associated with recent illness and/or food deprivation [5].

The causes of childhood malnutrition are diverse, multidimensional, and interrelated. An analytical framework suggested by the United Nations Children's Fund (UNICEF) categorizes the causes into (a) immediate causes: inadequate dietary intake and illness, (b) underlying causes: insufficient access to food in a household; inadequate health services and unhealthy environment; and inadequate care for children and women at the household level, and (c) basic causes: insufficient current and potential resources at societal level [6]. In Sub-Saharan Africa, various indicators of social economic status have been associated with children's nutritional status, such as maternal and paternal educational level, parental income, and family assets [7]–[9]. In addition, child nutrition outcomes in developing countries have been characterized by large rural-urban disparities over the last few decades [10].

In Ethiopia, child malnutrition continues to be a major public health problem. According to the Ethiopian National Demographic Health Survey (2011), the prevalence of both wasting and stunting in children under 5 years is very high (10% and 44% respectively) [11], while the situation in older children is not so well known [12], [13]. Furthermore, rural-urban disparities in child nutrition, as well as growing urbanization that results in increasing inequalities in urban areas, underlines the need to improve our knowledge of the main drivers of urban-rural differences [14].

The Amhara Region is one of the four primary agricultural regions in Ethiopia [15], and most households rely upon livestock and crop sales to generate cash income. This region, and especially the Tana Zuria Zone, has a moderate population density, fertile soils and good rainfall. For this reason, it is amongst the most food self-sufficient regions in the country [16]. Despite this good regional profile, other factors may be determining the high prevalence of infant malnutrition in this area [17]. According to the Food and Agriculture Organization of the United Nations (FAO), the four pillars of food security are food availability, stability of the food supply, food access and the utilization of food by the body [18]. In our context, availability is strongly affected by seasonality; many households are only able to produce sufficient food to meet their food requirements for less than six months of the year [19]. Food access may be affected by market conditions, but also by cultural and religious practices. For example, the high number of fasting days commemorated by the Ethiopian Orthodox Church, the main religion in the country, may have repercussions on the nutritional status of the community, particularly in rural Ethiopia [20].

Even if people get enough to eat, good nutrition requires access to a sufficient, supply of varied, safe and nutritious food to meet daily nutritional requirements [21]. Although diet diversity questionnaires are extensively used in Ethiopia to investigate relationships between food intake and nutritional status, there is limited knowledge of nutrition outcomes, dietary practices and socioeconomic factors among school-aged children in this specific context [17].

The present study aimed to [22] describe the prevalence of stunting and thinness, and their related factors, including dietary habits, and [23] document the differences in nutritional status across urban and rural areas accounting for household and individual characteristics in school-aged children in Libo Kemkem and Fogera, Amhara regional State, Ethiopia.

## Material and Methods

### Study area and population

The study was carried out during May–June 2009 in the districts (*woredas*) of Libo Kemkem and Fogera (Amhara regional state, Ethiopia). Libo Kemkem and Fogera woredas are located in the Tana Zuria Livelihood Zone, within the Amhara Regional State, northwestern Ethiopia at an altitude of 2,000 m above sea level. According to the 2009 census, the population was 198,374 and 226,595 for Libo Kemkem and Fogera, respectively.

These two districts are located in a black cotton clay soil flat plain. Temperatures are relatively high, but rainfall is unusually abundant, with a mean of 1173 mm per annum. Agricultural activities are dependent on a single rainy season (from June to September). Maize, barley and millet are the main food crops, while rice, vetch and chickpea are the main cash crops. Livestock holdings in sheep and cattle are relatively modest, but livestock and butter sales make a substantial complement to the predominant crop sales. The major hazards to crop production and livestock are pests, occasional flooding, and zoonosis such as anthrax, trypanosomiasis, pasteurellosis and black leg [16].

### Study design

This cross-sectional survey was part of a UBS Optimus Foundation funded project called Visceral Leishmaniasis (VL) and Malnutrition in Amhara State, Ethiopia. Among its specific objectives, the project aimed to characterize nutritional, immunological, and parasitological factors in school-aged children in the districts of Fogera and Libo Kemkem. Other methodological aspects have previously been published [24]–[26].

Sampling was carried out by multistage cluster survey. A total of 886 children aged 4 to 15 years were recruited. Primary sampling units were sub-districts (*kebeles*) with a high incidence of VL according to the 2008 register of the Addis Zemen VL Treatment Centre: one urban (Addis Zemen) and the rest rural: Bura, Yifag Akababi and Agita from Libo Kemkem, and Sifatra and Rib Gebriel from Fogera. Secondary sampling units were randomly selected villages (*gotts*) in each of the selected sub-districts. Third-stage sampling units were randomly selected households in each of the villages. All children with reported age between 4 and 15 years living in the household at the time of the survey were recruited. Sample size was calculated according to previous estimates of malnutrition for children under age 5 in the area and taking into account a design effect of 2, corresponding to the complex design.

### Data collection

All children were measured and weighed according to standard WHO procedures [27]. Weight was measured to the nearest 0.1 kg on a battery-powered digital scale (SECA 881©). Standing height was measured to the nearest 0.1 cm using a portable adult/infant measuring unit (PE-AIM-101©).

A pre-tested questionnaire translated into Amharic, the local language, was administered to the caretaker/head of household (HH) of each child in the study by trained medical personnel (nurses and health officers).

We asked about individual demographic characteristics, health status and behavior determinants. The following household variables were also recorded: household socio-demographic characteristics, person in charge of food preparation (PCFP), house construction material and assets (land and cultivation, domestic animal assets) and community variables. Dietary data was collected through a 24-hour diet recall.

### Statistical analysis

Stunting and thinness were the main outcomes of interest, defined as height-for-age z-score (HAZ) <−2 and BMI-for-age z-score (BAZ) <−2 respectively. The z-scores were calculated using the WHO 2007 reference (for children ≥5 years) and the WHO Growth Standards (for children <5 years old), both computed by WHO Anthro Plus software.

The dietary data collected through the 24-hour diet recall were computed into 9 food groups (1. Basic staples, 2. Vitamin A rich fruits and vegetables, 3. Other fruits, 4. Other vegetables, 5. Legumes and pulses, 6. Meat or Fish, 7. Oil, 8. Dairy and 9. Eggs) based on the FAO/FANTA Household Dietary Diversity Questionnaire and Guidelines [21].

Frequencies and percentages were used to summarize data and to explore the differences between rural and urban communities. These differences were assessed by Student's t-test and χ^2^ tests for continuous and categorical variables, respectively.

Bivariate analyses for thinness and stunting and their related factors were performed, with stratification by setting. Age and sex, considered biologically relevant, and all variables associated with each of the outcomes at the p<0.10 level were included in the multivariable analysis. Logistic regression models stratified by setting for stunting and thinness were obtained by using a manual backward stepwise procedure. P-values less than or equal to 0.05 were considered statistically significant.

Data analysis was performed using Anthro Plus v1.02 (WHO, Geneva, Switzerland) and SPSS version 18.0 (SPSS Inc., Chicago, Illinois, USA).

### Ethical considerations

The study was approved by the ethical review boards of the *Instituto de Salud Carlos III* (ISCIII), the Armauer Hansen Research Institute, and the Ethiopian National Ethical Review Committee. Support letters were obtained from the Amhara Regional State and the *woreda* health bureaus. Parents/guardians gave written informed consent before enrollment of their children in the study.

## Results

### Description of the sample

The study included a total of 886 children aged 4 to 15 years, of which 462 (52.0%) were males. Around 80% lived in the rural setting and 50.6% presented malnutrition. The prevalence of malnutrition was significantly higher in rural than in urban communities (53% and 42.1% respectively; p = 0.006).

The mean HAZ and BAZ for the overall study population were below the WHO references [28], −1.62 (SD = 1.34, range −6.12–5.74, p≤0.05) and −1.28 (SD = 1.00, range −4.95–3.82, p≤0.05), respectively.

Individual characteristics, including demographic data, nutritional status and diet habits are summarized in Table 1, disaggregated by rural and urban strata.

**Table 1:** Individual characteristics, behavioral determinants and dietary habits of school-aged children in rural and urban areas of at Libokemkem and Fogera districts, Ethiopia, May–June 2009.

CHARACTERISTICS	RURAL (n = 711)		URBAN (n = 178)		p value
	No.	(%)	No.	(%)	
**Sex (female)**	339	47,68	88	49,44	0,676
**Age group (≥10 years)**	259	36,43	58	32,58	0,193
**Stunting**	302	42,66	52	29,21	0,001
**Wasting**	153	21,61	37	20,79	0,450
**Splenomegaly**	34	4,79	1	0,56	0,004
**Fever in the last 15 days**	223	31,41	37	20,79	0,003
**Sleeps under a bed net**	292	41,13	120	67,42	<0.001
**Herds the cattle**	413	58,17	7	3,93	<0.001
**Consumption on day before survey of:**					
Food of animal source	129	18,14	114	64,04	<0.001
Basic staples	708	99,58	178	100	0,511
VitA rich fruits and vegetables	24	3,38	11	6,18	0,071
Other fruits	0	0	3	1,69	0,008
Other vegetables	53	7,45	22	12,36	0,029
Legumes and pulses	643	90,44	106	59,55	<0.001
Meat/fish	79	11,11	103	57,87	<0.001
Oil	644	90,58	166	93,26	0,164
Dairy	53	7,45	15	8,43	0,381
Eggs	5	0,7	5	2,81	0,032
5 or more food groups	28	3,94	16	8,99	0,007
4 or more food groups	116	16,32	71	39,89	<0.001
	**Mean**	**s.d.**	**Mean**	**s.d.**	**p value**
**Sum of food groups**	3,12	0,60	3,42	0,77	<0.001

There were several differences between children living in rural and urban settings regarding child health status, behavior and dietary habits. Children living in rural areas slept under a bed net less frequently than urban children (41.1% vs. 67.4% respectively, p<0.001). On the day before the survey, 18.1% of children in the rural setting had consumed food from animal sources vs. 64% of those in the urban setting.. The proportion of children who had consumed food from four or more food groups was 16.3% vs. 39.9%, respectively (p<0.001 for each comparison).

Household characteristics are summarized in Table 2. The proportion of rural households that owned land was 97.6% vs. 10.1% in urban households, while the corresponding figures for ownership of domestic animals or chickens were 96.3% vs. 36.5%, respectively (p<0.001 for each). The distribution of the rest of the socio-demographic and household variables but one (years of education of the HH) also differed significantly between urban and rural areas.

**Table 2:** Parental and household characteristics of school-aged children in rural and urban areas of Libo Kemkem and Fogera districts, Ethiopia, May–June 2009.

CHARACTERISTICS	RURAL (n = 711)		URBAN (n = 178)		p value
	No.	(%)	No.	(%)	
**Sex head of household (HH)**					
Female	52	7.31	75	42.13	<0.001
**Age HH**					
≥40 years	351	49.72	65	36.93	0.008
**HH literacy (read and write)**					
Yes	282	39.83	97	54.49	<0.001
**Person in charge of food preparation (PCFP)**					
Wife or HH(she)	690	97.05	144	80.9	<0.001
Other	21	2.95	34	19.1	
**Years of HH education**	**Mean**	**s.d.**	**Mean**	**s.d.**	**p value**
	2.70	12.6	4.27	4.92	0.106
**Years of education of the PCFP**					
	0.22	1	3.30	4.53	<0.001
**Number of people living in the house**					
	6.41	1.71	5.24	1.58	<0.001
**Number children in the house**					
	2.85	1.13	2.17	0.89	<0.001
					
**Does the household own land?**	**No.**	**(%)**	**No.**	**(%)**	**p value**
Yes	694	97.61	18	10.11	<0.001
**Have domestic animals or chickens?**					
Yes	685	96.34	65	36.52	<0.001
**Does the household….**					
**cultivate teff?**					
Yes	481	67.65	4	2.25	<0.001
**cultivate rice?**					
Yes	231	32.49	2	1.12	<0.001
**cultivate millet?**					
Yes	70	9.85	5	2.81	<0.001
**cultivate beans?**					
Yes	22	3.09	1	0.56	0.038
**consume products from their own cattle?**					
Do not consume own cattle products	224	31.68	0		
Consume own cattle products	419	59.26	11	6.29	<0.001
Do not have cattle	64	9.05	164	93.71	
**consume products from their own goats?**					
Do not consume own goat products	35	4.96	0		
Consume own goat products	34	4.82	7	3.93	0.008
Do not have goats	637	90.23	171	96.07	
**consume products from their own sheep?**					
Do not consume own sheep products	62	8.81	2	1.12	
Consume own sheep products	90	12.78	6	3.37	<0.001
Do not have sheep	552	78.41	170	95.51	
**consume products from their own chickens?**					
Do not consume own chicken products	80	11.28	2	1.12	
Consume own chicken products	343	48.38	47	26.40	<0.001
Do not have chickens	286	40.34	129	72.47	

### Chronic malnutrition

The prevalence of stunting was 42.7% in the rural setting and 29.2% in the urban setting (p = 0.001).

#### Chronic malnutrition in rural communities

In the bivariate analysis (Table S1), the prevalence of chronic malnutrition was similar in males and females, however it was significantly higher inchildren age 10 and over than in those under 10 years of age (OR: 2.28; 95% CI: 1.66–3.11) in the rural group. Stunting was significantly more frequent in children with a previous history of splenomegaly (OR: 2.26; 95% CI: 1.11–4.59) or fever (OR: 1.59; 95% CI: 1.15–2.18), and less common in those who slept under a bed net (OR: 0.68; 95% CI: 0.50–0.92) or herded the cattle (OR: 0.70; 95% CI: 0.52–0.95). Regarding dietary habits in rural communities, the consumption of vitamin A rich fruits and vegetables and the consumption of any meat or fish the day before the survey were associated with a lower prevalence of chronic malnutrition (OR: 0.34 (95% CI: 0.13–0.93) and OR: 0.59 (95% CI: 0.36–0.97), respectively).

Results from the multivariable logistic analysis are presented in Table 3. The difference between the two age groups remained significant in the final model (OR: 3.12; 95% CI: 2.15–4.51), showing that the prevalence of chronic malnutrition was higher in children age 10 and over. Children with fever in the two weeks prior to the survey were 1.62 times more likely to be stunted (95% CI: 1.23–2.32). Herding cattle (OR: 0.43; 95% CI: 0.30–0.63) and the consumption of any food from animal sources (OR: 0.51; 95% CI: 0.29–0.91) showed a significant negative association with stunting. With regard to household socio demographic characteristics, children from rural areas were 2.97 times more likely to have chronic malnutrition if the HH was female (95%CI: 1.47–5.98). The number of people living in the house was positively associated with the prevalence of stunting (p = 0.042), while millet production (OR: 0.50; 95% CI: 0.27–0.93) and consumption of products from the family's own cattle (OR: 0.67; 95% CI: 0.46–0.96) were negatively associated.

**Table 3:** Multivariable logistic regression analysis of stunting in school-aged children stratified by setting in Libo Kemkem and Fogera districts, Ethiopia, May–July 2009.

VARIABLES		RURAL (N = 711)				URBAN (N = 178)			
		n (%)	AOR	95% CI	p value	n (%)	AOR	95% CI	p value
**CHILD CHARACTERISTICS**									
**Sex**	**male**	166 (44.86)	1			27 (30.00)	1		
	**female**	136 (40.24)	0.78	(0.55–1.10)	0.160	25 (28.41)	1.29	(0.56–2.96)	0.554
**Age group**	**<10 years**	159 (35.24)	1			24 (20.01)	1		
	**≥10 years**	143 (55.26)	3.12	(2.15–4.51)	0.000	28 (48.39)	4.62	(2.09–10.21)	0.000
**Fever in the last 15 days?**	**No**	112 (50.45)	1			13 (35.14)	1		
	**Yes**	190 (39.09)	1.62	(1.23–2.32)	0.009	39 (27.66)	1.80	(0.73–4.47)	0.204
**Does the child herd the cattle?**	**No**	141 (47.64)	1			39 (29.24)	1		
	**Yes**	160 (38.93)	0.43	(0.30–0.63)	0.000	13 (28.57)	0.75	(0.07–7.71)	0.811
**DOES THE CHILD CONSUME…**									
**Any food from animal sources** *	**No**	254 (43.87)	1			20 (31.25)	1		
	**Yes**	48 (37.21)	0.51	(0.29–0.91)	0.022	32 (28.07)	0.72	(0.31–1.71)	0.463
**vit A rich fruits and vegetables** *	**No**	297 (43.42)	1			48 (28.74)	1		
	**Yes**	5 (20.83)	0.29	(0.83–1.04)	0.057	4 (36.36)	1.32	(0.29–5.99)	0.717
**Other vegetables** *	**No**	284 (43.36)	1			42 (26.92)	1		
	**Yes**	18 (33.96)	1.08	(0.49–2.38)	0.855	10 (45.45)	3.00	(0.97–9.38)	0.058
**HOUSEHOLD AND LAND PRODUCTION**									
**Sex of head of household**	**Male**	267 (40.70)	1			29 (28.16)	1		
	**Female**	35 (67.31)	2.97	(1.47–5.98)	0.002	23 (30.67)	0.66	(0.27–1.64)	0.370
**Age of head of household**	**<40 years**	151 (42.66)	1			37 (28.16)	1		
	**≥40 years**	147 (42.14)	0.73	(0.50–1.06)	0.097	23 (30.67)	0.43	(0.17–1.06)	0.069
**Years of school-person in charge of food preparation**		**Mean (sd)**	**AOR**	**95% CI**	**p value**	**Mean (sd)**	**AOR**	**95% CI**	**p value**
		0.16 (0.86)	0.84	(0.70–1.01)	0.065	2.19 (4.0)	0.88	(0.79–0.97)	0.014
**Number of people living in the house**									
		6.43 (1.85)	1.12	(1.01–1.25)	0.042	5.08 (1.41)	0.84	(0.63–1.13)	0.260
		**n (%)**	**AOR**	**95% CI**	**p value**	**n (%)**	**AOR**	**95% CI**	**p value**
**Does the family cultivate millet?**	**No**	280 (43.82)	1			50 (28.90)	1		
	**Yes**	22 (31.88)	0.50	(0.27–0.93)	0.029	2 (40.00)	2.35	(0.27–20.19)	0.435
**Does the family consume products from their own cattle?**	**Do not consume own products**	39 (60.94)	1			47 (28.66)	1		
	**Consume own products**	107 (48.20)	0.67	(0.46–0.96)	0.030	0	-	-	N.A.
	**Do not have cattle**	154 (36.84)	1.36	(0.72–2.56)	0.348	3 (27.27)	1.07	(0.11–9.23)	0.989
**Does the family consume products from their own goats?**	**Do not consume own products**	277 (43.69)	1			52 (30.41)	1		
	**Consume own products**	16 (45.71)	0.37	(0.12–1.16)	0.090	0	-	-	N.A.
	**Do not have goats**	8 (23.53)	0.92	(0.43–1.96)	0.833	0	-	-	N.A.

*day before the survey.

#### Chronic malnutrition in urban communities

In the urban setting, the significant age differences (OR: 3.73; 95% CI: 1.89–7.39) found in the bivariate analysis occurred in the same direction as in the rural area (Table S1). Furthermore, the prevalence of stunting was significantly higher among children living in a house where the HH (OR: 1.58; 95% CI: 1.00–2.53) or the PCFP (OR: 1.60; 95% CI: 0.99–2.66) had not attended school.

Table 3 summarizes the results adjusted by logistic regression. Older children were more prone to stunting than younger children (OR: 4.62; 95% CI: 2.09–10.21). Years of school attendance of the PCFP was negatively associated with chronic malnutrition among children living in urban communities (OR: 0.88; 95% CI: 0.79–0.97).

### Acute malnutrition

The prevalence of thinness was 21.6% in the rural setting and 20.8% in the urban setting (p>0.05).

#### Acute malnutrition in rural communities

In the bivariate analysis (Table S2), the prevalence of acute malnutrition was higher in children age 10 and over (OR: 4.74; 95% CI: 3.24–6.94) and among boys (OR: 1.58; 95% CI: 1.18–2.12). Children from rural communities who herded the cattle were 2.43 times more likely to be thin (95%CI: 1.63–3.61) than those who did not. The number of children living in the house (p = 0.007) and teff cultivation (OR: 1.53; 95% CI: 1.02–2.30) were positively associated with higher prevalence of thinness, while living with a HH under 40 years old and rice farming showed an inverse association with thinness (OR: 0.75; 95% CI: 0.56–0.99 and 0.63; 95% CI: 0.42–0.95, respectively).

After adjusting for socio demographic and household characteristics in the model, sex differences lost significance, and the relationship between thinness and age was slightly weakened (OR: 4.11; 95% CI: 2.74–6.16) (Table 4). Children from rural communities were significantly less likely to be thin if the HH was female (OR: 0.40; 95% CI: 0.16–0.70). The number of children living in the house showed a positive relationship with thinness in this setting (p = 0.027), while children from households that cultivate rice were less likely to be thin (OR: 0.64; 95% CI: 0.41–0.99).

**Table 4:** Multivariable logistic regression analysis of thinness in school-aged children, stratified by setting in Libo Kemkem and Fogera districts, Ethiopia, May–July 2009.

VARIABLES		RURAL (N = 711)				URBAN (N = 178)			
		n (%)	Adjusted OR	95% CI	p value	n (%)	Adjusted OR	95% CI	p value
**CHILD CHARACTERISTICS**									
**Sex**	**male**	97 (26.22)	1			21 (23.33)	1		
	**female**	56 (16.57)	0.69	(0.46–1.03)	0.073	16 (18.18)	0.86	(0.39–1.92)	0.717
**Age group**	**<10 years**	53 (11.83)	1			18 (15.02)	1		
	**≥10 years**	100 (38.84)	4.11	(2.74–6.16)	0.000	19 (32.81)	3.67	(1.63–8.30)	0.002
** Does the child herd the cattle?**	**No**	40 (13.51)	1			36 (21.05)	1		
	**Yes**	113 (27.49)	1.50	(0.96–2.36)	0.076	1 (14.29)	0.55	(0.06–5.03)	0.598
**DOES THE CHILD CONSUME…**									
**Any food from animal sources**	**No**	129 (22.28)	1			18 (28.13)	1		
	**Yes**	24 (18.60)	0.83	(0.49–1.41)	0.493	19 (16.67)	0.26	(0.10–0.67)	0.005
**HOUSEHOLD AND LAND PRODUCTION**									
**Sex of head of household**	**Male**	146 (22.26)	1			23 (22.33)	1		
	**Female**	7 (13.46)	0.40	(0.16–0.70)	0.043	14 (18.67)	0.67	(0.26–1.72)	0.407
**Literacy of head of household (can read and write)**	**No**	93 (21.83)	1			25 (25.77)	1		
	**Yes**	60 (21.51)	1.39	(0.91–2.11)	0.127	12 (14.81)	0.24	(0.09–0.65)	0.005
**Number of people living in the house**		**Mean (sd)**	**AOR**	**95% CI**	**p value**	**Mean (sd)**	**AOR**	**95% CI**	**p value**
		6.45 (1.68)	0.87	(0.75–1.00)	0.054	5.14 (1.72)	0.87	(0.64–1.20)	0.403
**Number of children in the house**									
		3.06 (1.14)	1.28	(1.03–1.60)	0.027	2.19 (0.94)	1.02	(0.61–1.70)	0.955
		**n (%)**	**AOR**	**95% CI**	**p value**	**n (%)**	**AOR**	**95% CI**	**p value**
**Does the family cultivate rice?**	**No**	115 (24.01)	1			36 (20.45)	1		
	**Yes**	38 (16.59)	0.64	(0.41–0.99)	0.045	1 (50.00)	3.26	(0.15–72.94)	0.456

#### Acute malnutrition in urban communities

In the bivariate analysis (Table S2), no significant associations with thinness were found apart from age group (OR: 2.76; 95% CI: 1.13–5.80).

After adjusting the analysis, age group remained significantly related to thinness (OR: 3.67; 95% CI: 1.63–8.30). Food consumption from animal sources on the day before the survey was inversely associated with acute malnutrition (OR: 0.26; 95% CI: 0.10–0.67) and thinness prevalence was lower among in households headed by literate persons (OR: 0.24; 95% CI: 0.09–0.65) (Table 4).

## Discussion

Our study shows that there is a high prevalence of stunting (39.8%) and thinness (21.4%) among school-aged children in Libo Kemkem and Fogera regions of Ethiopia. The prevalence of stunting was significantly higher in rural areas (42.7% vs. 29.2%), but no significant differences were observed for thinness. These results are similar to those observed in other developing countries [29]. Various intermediate and distal factors like age, consumption of food from animal sources and family size were associated with both types of malnutrition in one or both settings. Other determinants such as years of school attendance of the PCFP and consumption of the family's own cattle products were related to only one kind of malnutrition. Although malnutrition among pre-school children has been well documented in Ethiopia [5], [30], [31], to our knowledge this is the first research to assess factors related to acute and chronic malnutrition stratified by setting in school aged children. These results may assist stakeholders in planning and undertaking contextual and evidence-based policy initiatives.

We found that the probability of a child being malnourished increases with age. Age-group differences were significant in both strata for stunting and thinness. No sex differences were found in either strata. As children mature, household socioeconomic characteristics may emerge in conjunction with behavioral and biological variables as important risk factors [32].

### Chronic malnutrition

The prevalence of stunting in rural areas in our study was higher (42.7%) than that found in a study conducted in the same age group in rural settings of Fogera in 2012 (30.7%) [33]. Our study was carried out in 2009, which may partially explain this difference due to possible improvements in local conditions; another reason may be that children in the Fogera study had to be enrolled in school in order to participate, which could result in selection bias. In addition, our sampling was done in sub-districts with a high incidence of VL, a characteristic that may be associated with fewer resources and worse health status in children. The prevalence of chronic malnutrition in the urban area (29.2%) could not be compared to previous data due to the lack of research targeting this particular age group in this setting.

In rural communities, the setting with the highest stunting prevalence, we found several factors associated with chronic malnutrition: age group, fever in the previous 15 days, herding the cattle, consumption of any food from animal sources, sex of the HH, family size, cultivation of millet and consumption of the family's own cattle products. These factors should be considered when targeting chronic malnutrition in this region.

Children who had fever in the previous 15 days were 62% more likely to be stunted than those who did not. Infection and malnutrition are intricately linked through extensive, synergistic, antagonistic, and cyclical interactions [34], [35]. Although our study area is known to be a low endemic area for malaria and leishmaniasis [25], [36], other specific infections associated with malnutrition (such as chronic parasitic infestations) are highly prevalent [37], [38]. Infectious diseases manifested in the form of fever affect both dietary intake and utilization, which may affect child growth. Not having empirical data on specific infections is a limitation, but we considered fever in the previous 15 days could act as a proxy for non-specific infection. On the other hand, stunted growth and related immunosuppression may lead to intermittent fever [39].

In our research, children who herd the cattle were less likely to be stunted. This could be explained by better activity levels in non-stunted children, given that stunted children show behavioral differences in early childhood including apathy and reduced activity, play and exploration [40].

Finally, children from households with millet farming and from families who consume their own cattle products were less likely to be stunted in rural communities. Although most rural families own land and animals (97.6% and 96.3% respectively), we observed that they do not consume their own products as often. Only 11.1% of the children had eaten any meat or fish over the last 24 hours, while 99.6% and 90.4%, respectively, had consumed basic staples and legumes and/or pulses respectively. In Ethiopia, child diet is based mainly based on plant foods like the traditional Ethiopian staple food called “*injera*”, a yeast-risen flatbread made of a blend of cereals, usually served with legumes or pulses. This may not provide all the nutritional requirements of children [41]. We are aware that market access in this livelihood zone is poor; moreover what little trade interaction exists is restricted to cash crops that are attractive for their high price (i.e. rice and teff), while other essential crops cultivated mainly for personal consumption face disincentives [16], [42].

In urban communities, age group and years of school of the PCFP were significantly associated with chronic malnutrition in children. This educational factor may operate indirectly to affect children's nutritional status by determining the quality of the child's diet, care and physical environment [30]. The level of education of the PCFP may have a positive impact on his/her knowledge on food facilities, controlling contamination, time and temperature parameters for controlling pathogens, and advice on good dietary habits [43].

### Acute malnutrition

The prevalence of acute malnutrition in the rural settings of Fogera and Libo Kemkem was 21.6%. The prevalence of underweight found in the study conducted in Fogera was 37.2% [33]. These results cannot be directly compared as different anthropometric indices were used. And again, we did not find any data on thinness to compare with our results in the urban population.

In rural settings, age group, sex of the HH, number of children in the house and rice cultivation were factors associated with thinness. The number of children in a household and the prevalence of thinness were positively associated. Larger family size may put children at higher risk for acute malnutrition, which could be due to the imbalance between family size and resources [44].

Those whose families cultivate rice were less likely to be thin in rural communities. In this zone, rice production might be acting as a proxy for better socioeconomic status, as rice consumption is relatively recent but is one of the main cash crops in the area [16]. The low consumption of animal source foods and its association with acute malnutrition has been previously identified as a major contributing factor to delayed growth in children [45] and suboptimal dietary practices among adolescents in Ethiopia [17].

In urban areas, children with a literate HH were 4 times less likely to be thin than those living in houses headed by illiterate adults. Some studies have shown that parental education is associated with more efficient management of limited household resources, improved utilization of available health care services, and better health-promoting behaviors, all of which are associated with better child nutrition [46], [47]. This result is similar to what we previously observed for stunting and PCFP years of education in the urban setting. A possible explanation could be the existence of an educational gap in urban but not in rural areas.

### Stunting versus thinness: associated factors

Clear differences among risk factors for stunting (Table 3) and thinness (Table 4) emerged from this study. The literature on the causes of stunting is vast, and conventional thinking is summarized in the *Lancet* series on maternal and child under-nutrition [48]. Recognized causal factors include prenatal and postnatal periods. Stunting is seen as closely tied to poverty and access to services. Less knowledge is available on risk factors for thinness [1]. In our research, risk factors for chronic malnutrition encompass a wide range of variables.. The relatively consistent pattern of related factors for stunting suggests that continued exposure to adverse conditions retards children's linear growth. Conversely, the greater diversity observed in the factors associated with thinness is consistent with the fact that a relatively short period of risk exposure can precipitate its onset in children [32].

In rural communities, children from male-headed households were more likely to be thin than children from female-headed households (p = 0.043), while stunting was significantly more frequent in female-headed households (p = 0.002). The disparate sample size in rural and urban areas may have influenced these results. However, the result in the rural area is consistent with the study conducted in North Ethiopia by Haidar et al. [49]. This study found a significantly higher proportion of stunted and underweight pre-school children in female headed-households, whereas the prevalence of thinness was similar [48]–[50]. Women who are single HH may be removed from their support structures and may face constraints in accessing services, including food, as a result of insecurity, cultural discrimination and limited mobility [48], [50]. This situation may have a long-term impact in child nutrition.

### Rural versus urban: associated factors

Ethiopia remains one of the least urbanized countries in the world [42]. Globally, malnutrition is less common in urban areas [2], [31]. We found that malnutrition in rural communities was associated with food habits and the lack of material resources whereas in the urban area, it was better predicted by socio demographic factors. Inequalities in child health outcomes are known to vary between rural and urban areas, and are often due to unequal allocation of resources [51].

Significant urban-rural differences remained in the multivariable model. This shows that even in the presence of important individual factors and socioeconomic variables, area of residence is still a predictor of children's nutritional status. Our results highlight the need to stratify data when rural and urban communities are targeted in nutritional research in this kind of context.

### Limitations

The present study was conducted in two single districts in Ethiopia, thus, the findings may not be generalizable to a larger population. Additionally, the cross-sectional nature of this data does not allow us to examine causality in the relationship between malnutrition and diverse risk factors. Seasonality should be given special attention; the season of the year has a significant effect not only on food security and nutritional status, but also on patterns and trends of infectious disease incidence. Therefore, consecutive measurements are desirable..

This research is part of a project which aimed to characterize nutritional, immunological, and parasitological aspects in school-aged children from urban and rural villages with a high incidence of VL in 2005–07. However, the VL prevalence found in the study was very low [25], and no association was found between nutritional status and asymptomatic infection [24]. Therefore, we are confident that this limitation does not alter our general conclusions.

## Conclusions

Our findings suggest that improving food availability is a necessary but not sufficient condition to improve the nutritional status of school-aged children in this region. Especially in rural areas, the challenge will be for health and development extension workers to build on this knowledge through educational campaigns when advising households about balanced diet, food production and consumption, and hygienic behavior. It is also important to emphasize that nutritional programs should not be biased towards rural areas at the cost of excluding the urban poor.

To effectively tackle malnutrition, nutritional programs should be oriented to the local needs. Our findings can be used to help policy makers plan and undertake regional initiatives to streamline recommendations.

## Supporting Information

Table S1 Factors related to stunting in school-aged children by setting in Libokemkem and Fogera districts, Ethiopia, May–June 2009. Bivariate analysis.
(DOCX)

Table S2 Factors related to thinness in school-aged children by setting in Libokemkem and Fogera districts, Ethiopia, May–June 2009. Bivariate analysis.
(DOCX)
