# Supplementary material for: Cross-Sectional Study of Malnutrition and Associated Factors among School Aged Children in Rural and Urban Settings of Fogera and Libo Kemkem Districts, Ethiopia
Source: PLoS One. 2014 Sep 29;9(9):e105880. doi: 10.1371/journal.pone.0105880 (PMC4179248; doi:10.1371/journal.pone.0105880)
Supplement: Table S1 — Factors related to stunting in school-aged children by setting in Libokemkem and Fogera districts, Ethiopia, May–June 2009. Bivariate analysis. (DOCX) [file pone.0105880.s001.docx]

| **Table S1. Factors related to stunting in school-aged children by setting in Libokemkem and Fogera districts, Ethiopia, May–June 2009. Bivariate analysis.** | | | | | | |
| --- | --- | --- | --- | --- | --- | --- |
| **Variable** | **RURAL (n=711)** | | | **URBAN (n=178)** | | |
|  | **N** | **%** | **UOR (95% CI)** | **N** | **%** | **UOR (95% CI)** |
| **No. of subjects** | 302 | 42.48 |  | 52 | 29.21 |  |
| **Sex** |  |  |  |  |  |  |
| Male | 166 | 44.62 | 1.12 (0.94-1.33)* | 27 | 30.00 | 1.06 (0.67-1.67) |
| Female | 136 | 40.12 |  | 25 | 28.41 |  |
| **Age group** |  |  |  |  |  |  |
| < 10 years | 159 | 35.18 | 2.28 (1.67-3.11)** | 24 | 20.00 | 3.73 (1.89-7.39)** |
| ≥ 10 years | 143 | 55.21 |  | 28 | 48.28 |  |
| **Child has splenomegaly?** |  |  |  |  |  |  |
| Yes | 21 | 61.76 | 2.26 (1.11-4.59)** | 1ⱡ | 50.00 | 1.18 (0.10-13.28) |
| No | 281 | 41.57 |  | 53 | 29.50 |  |
| **Fever in the last 15 days?** |  |  |  |  |  |  |
| Yes | 112 | 50.22 | 1.59 (1.15-2.18)** | 13 | 35.10 | 1.42 (0.66-3.06) |
| No | 190 | 39.01 |  | 39 | 27.70 |  |
| **Child sleeps under a bed net?** |  |  |  |  |  |  |
| No | 194 | 40.76 | 0.68 (0.50-0.92)** | 18 | 31.03 | 0.88 (0.44-1.74) |
| Yes | 107 | 25.97 |  | 34 | 28.33 |  |
| **Child herds the cattle?** |  |  |  |  |  |  |
| No | 141 | 47.60 | 0.70 (0.52-0.95)** | 39 | 29.20 | 0.97 (0.18-5.16) |
| Yes | 160 | 38.90 |  | 13 | 28.60 |  |
| **Consumption on day before survey of** |  |  |  |  |  |  |
| Food from animal source |  |  |  |  |  |  |
| No | 254 | 43.87 | 0.76 (0.51-1,12)* | 20 | 31.25 | 0.86 (0.44-1.67) |
| Yes | 48 | 37.21 |  | 32 | 28.07 |  |
| **Basic staples** |  |  |  |  |  |  |
| No | 2 | 66.67 | 0.37 (0.03-4.10) | 0 | 0.00 | N.A. |
| Yes | 300 | 42.55 |  | 52 | 29.20 |  |
| **VitA rich fruits and vegetables** |  |  |  |  |  |  |
| No | 297 | 43.42 | 0.34 (0.13-0.93)** | 48 | 28.74 | 1.42 (0.40-5.06) |
| Yes | 5 | 20.83 |  | 4 | 36.36 |  |
| **Other fruits** |  |  |  |  |  |  |
| No | 302 | 42.66 | N.A. | 52 | 29.71 | N.A. |
| Yes | 0 | 0.00 |  | 0 | 0.00 |  |
| **Other vegetables** |  |  |  |  |  |  |
| No | 284 | 43.36 | 0.67 (0.37-1.21)* | 42 | 26.92 | 2.26 (0.91-5.62)* |
| Yes | 18 | 33.96 |  | 10 | 45.45 |  |
| **Legumes and pulses** |  |  |  |  |  |  |
| No | 22 | 32.35 | 1.63 (0.96-2.77)* | 17 | 23.61 | 1.60 (0.81-3.14) |
| Yes | 280 | 43.75 |  | 35 | 33.02 |  |
| **Meat/fish** |  |  |  |  |  |  |
| No | 277 | 44.04 | 0.59 (0.36-0.97)** | 24 | 32.00 | 0.79 (0.41-1.52) |
| Yes | 25 | 31.65 |  | 28 | 27.18 |  |
| **Oil** |  |  |  |  |  |  |
| No | 34 | 51.51 | 0.67 (0.41-1.12)* | 3 | 25.00 | 1.26 (0.33-4.84) |
| Yes | 268 | 41.74 |  | 49 | 29.52 |  |
| **Dairy** |  |  |  |  |  |  |
| No | 278 | 42.44 | 1,12 (0.64-1.97) | 45 | 27.61 | 2.29 (0.79-6.70)* |
| Yes | 24 | 45.28 |  | 7 | 46.67 |  |
| **Eggs** |  |  |  |  |  |  |
| No | 301 | 42.82 | 0.33 (0.04-3.00) | 51 | 29.48 | 0.60 (0.07-5.48) |
| Yes | 1 | 20.00 |  | 1 | 20.00 |  |
| **5 or more food groups** |  |  |  |  |  |  |
| No | 261 | 44.09 | 1.25 (0.96-1.62)* | 24 | 22.43 | 0.57 (0.36-0.90)** |
| Yes | 41 | 35.53 |  | 28 | 39.44 |  |
| **4 or more food groups** |  |  |  |  |  |  |
| No | 298 | 43.82 | 3.07 (1.23-7.63)** | 48 | 29.63 | 1.19 (0.49-2.86) |
| Yes | 4 | 14.29 |  | 4 | 25.00 |  |
| **HOUSEHOLD VARIABLES** | | | | | | |
| **Sex head of household(HH)** |  |  |  |  |  |  |
| Male | 267 | 40.70 | 0.61 (0.49-0.75)** | 29 | 28.16 | 0.92 (0.58-1.45) |
| Female | 35 | 67.31 |  | 23 | 30.67 |  |
| **Age HH** |  |  |  |  |  |  |
| < 40 years | 151 | 42.66 | 1.01 (0.85-1.20) | 37 | 33.33 | 1.44 (0.86-2.42)* |
| ≥ 40 years | 147 | 42.12 |  | 15 | 23.08 |  |
| **HH literacy (read and write)** |  |  |  |  |  |  |
| Yes | 115 | 41.22 | 0.95 (0.80-1.13) | 22 | 22.68 | 0.61 (0.39-0.97)** |
| No | 185 | 43.43 |  | 30 | 37.04 |  |
| **Person in charge of food preparation (PCFP)** |  |  |  |  |  |  |
| Wife or HH(she) | 294 | 42.79 | 1.12 (0.65-1.95) | 46 | 31.94 | 1.81 (0.84-3.89)* |
| Other | 8 | 38.09 |  | 6 | 17.65 |  |
| **Years of education of the PCFP** | **Mean** | **s.d.** | **p value** | **Mean** | **s.d.** | **p value** |
|  | 1,07 | 2.29 | 0.767 | 3,48 | 4.74 | 0.172 |
| **Number of people living in the house** |  |  |  |  |  |  |
|  | 6,43 | 1.85 | 0.806 | 5,08 | 1.41 | 0.389 |
| **Number of children in the house** |  |  |  |  |  |  |
|  | 2,82 | 1.14 | 0.674 | 2,29 | 0.98 | 0.252 |
| **DOES THE HOUSEHOLD….** |  |  |  |  |  |  |
| **own land?** |  |  |  |  |  |  |
| No | 7 | 41.18 | 0.97 (0.54-1.71) | 45 | 28.13 | 0.72 (0.39-1.36) |
| Yes | 295 | 42.69 |  | 7 | 38.89 |  |
| **have domestic animals or chicken?** |  |  |  |  |  |  |
| No | 15 | 57.69 | 1.37 (0.98-1.93)* | 30 | 26.55 | 0.78 (0.50-1.24) |
| Yes | 287 | 42.08 |  | 22 | 33.85 |  |
| **cultivate teff?** |  |  |  |  |  |  |
| No | 100 | 43.48 | 0.95 (0.69-1.31) | 50 | 28.74 | 2.48 (0.34-18.09) |
| Yes | 202 | 42.26 |  | 2 | 50.00 |  |
| **cultivate rice?** |  |  |  |  |  |  |
| No | 196 | 40.92 | 1.24 (0.91-1.71)* | 51 | 28.97 | 2.45 (0.15-39.94) |
| Yes | 106 | 46.29 |  | 1 | 50.00 |  |
| **cultivate millet?** |  |  |  |  |  |  |
| No | 280 | 43.82 | 1.37 (0.96-1.96)* | 50 | 28.90 | 0.72 (0.24-2.17) |
| Yes | 22 | 31.88 |  | 2 | 40.00 |  |
| **cultivate beans?** |  |  |  |  |  |  |
| No | 297 | 43.29 | 1.91 (0.88-4.14)* | 52 | 29.38 | N.A. |
| Yes | 5 | 22.73 |  | 0 | 0.00 |  |
| **consume products from their own cattle?** |  |  |  |  |  |  |
| Do not consume own cattle products | 39 | 60.94 | 1 | 47 | 28.66 | 1 |
| Consume own cattle products | 107 | 48.20 | 0.60 (0.34-1.05)* | 0 | 0.00 | N.A. |
| Do not have cattle | 154 | 36.84 | 0.37 (0.22-0.64)** | 3 | 27.27 | 0.93 (0.24-3.67) |
| **consume products from own goats?** |  |  |  |  |  |  |
| Do not consume own goat products | 277 | 43.69 | 1 | 52 | 30.41 | 1 |
| Consume own goat products | 16 | 45.71 | 1.09 (0.55-2.15) | 0 | 0.00 | N.A. |
| Do not have goat | 8 | 23.53 | 0.40 (0.18-0.89)** | 0 | 0.00 | N.A. |
| **consume products from their own sheep?** |  |  |  |  |  |  |
| Do not consume own sheep products | 235 | 4.73 | 1 | 51 | 30.00 | 1 |
| Consume own sheep products | 28 | 45.16 | 1.10 (0.65-1.87) | 0 | 0.00 | N.A. |
| Do not have sheep | 39 | 43.82 | 1.05 (0.67-1.64) | 1 | 16.70 | 0.47 (0.05-4.10) |
| **consume products from their own chickens?** |  |  |  |  |  |  |
| Do not consume own chicken products | 120 | 41.96 | 1 | 34 | 26.36 | 1 |
| Consume own chicken products | 34 | 43.04 | 1.04 (0.63-1.73) | 2 | 100.00 | N.A. |
| Do not have chicken | 147 | 43.11 | 1.05 (0.76-1.44) | 16 | 34.04 | 1.44 (0.70-2.96) |
| *p<0.10; ** p<0.05 | | | | | | |
