# Supplementary material for: Cross-Sectional Study of Malnutrition and Associated Factors among School Aged Children in Rural and Urban Settings of Fogera and Libo Kemkem Districts, Ethiopia
Source: PLoS One. 2014 Sep 29;9(9):e105880. doi: 10.1371/journal.pone.0105880 (PMC4179248; doi:10.1371/journal.pone.0105880)
Supplement: Table S2 — Factors related to thinness in school-aged children by setting in Libokemkem and Fogera districts, Ethiopia, May–June 2009. Bivariate analysis. (DOCX) [file pone.0105880.s002.docx]

| **Table S2. Factors related to thinness in school-aged children by setting in Libokemkem and Fogera districts, Ethiopia, May–June 2009. Bivariate analysis.** | | | | | | |
| --- | --- | --- | --- | --- | --- | --- |
| **Variable** | **RURAL (n=711)** | | | **URBAN (n=178)** | | |
|  | **N** | **%** | **UOR (95% CI)** | **N** | **%** | **UOR (95% CI)** |
| **No. of subjects** | 153 | 21.52 |  | 37 | 20.79 |  |
| **Sex** |  |  |  |  |  |  |
| Male | 97 | 26.22 | 1.58 (1.18-2.12)** | 21 | 23.30 | 1.28 (0.72-2.29) |
| Female | 56 | 16.57 |  | 16 | 18,20 |  |
| **Age group** |  |  |  |  |  |  |
| < 10 years | 53 | 11.78 | 4.74 (3.24-6.94)** | 18 | 15.00 | 2.76 (1.13-5.80)** |
| ≥ 10 years | 100 | 38.78 |  | 19 | 32.80 |  |
| **Child has splenomegaly?** |  |  |  |  |  |  |
| Yes | 11 | 32.40 | 1.79 (0.85-3.76)* | 1ⱡ | 50.00 | 1.84 (0.16-20.86) |
| No | 142 | 21.10 |  | 38 | 21.00 |  |
| **Fever in the last 15 days?** |  |  |  |  |  |  |
| Yes | 53 | 23.90 | 1.21 (0.83-1.77) | 8 | 21.60 | 1.07 (0.44-2,58) |
| No | 100 | 20.60 |  | 29 | 20.60 |  |
| **Child sleeps under a bed net?** |  |  |  |  |  |  |
| No | 89 | 21,30 | 1.05 (0.73-1.51) | 10 | 17.24 | 1.39 (0.62-3.12) |
| Yes | 64 | 22,10 |  | 27 | 22.50 |  |
| **Child herds the cattle?** |  |  |  |  |  |  |
| No | 40 | 13.50 | 2.43 (1.63-3.61)** | 36 | 21.10 | 0.63 (0.07-5.36) |
| Yes | 113 | 27.50 |  | 1 | 14.30 |  |
| **Consumption on day before survey of** |  |  |  |  |  |  |
| Food from animal source |  |  |  |  |  |  |
| No | 129 | 22.28 | 0.80 (0.49-1.30) | 18 | 28.13 | 0.51 (0.25-1.06)* |
| Yes | 24 | 18.60 |  | 19 | 16.67 |  |
| Basic staples |  |  |  |  |  |  |
| No | 0 | 0.00 | N.A. | 0 | 0.00 | N.A. |
| Yes | 153 | 21.70 |  | 37 | 20.80 |  |
| VitA rich fruits and vegetables |  |  |  |  |  |  |
| No | 149 | 21.78 | 0.72 (0.24-2.13) | 36 | 21.56 | 0.36 (0.05-2.94) |
| Yes | 4 | 16.67 |  | 1 | 9.09 |  |
| Other fruits |  |  |  |  |  |  |
| No | 153 | 21.61 | N.A. | 37 | 21.14 | N.A. |
| Yes | 0 | 0.00 |  | 0 | 0.00 |  |
| Other vegetables |  |  |  |  |  |  |
| No | 141 | 21.53 | 1.07 (0.55-2.08) | 30 | 19.23 | 1.96 (0.73-5.23) |
| Yes | 12 | 22.64 |  | 7 | 31.82 |  |
| Legumes and pulses |  |  |  |  |  |  |
| No | 13 | 19.12 | 1.18(0.63-2.23) | 14 | 19.44 | 1.15(0.55-2.42) |
| Yes | 140 | 21.88 |  | 23 | 21.70 |  |
| Meat/fish |  |  |  |  |  |  |
| No | 138 | 21.94 | 0.83 (0.46-1.51) | 18 | 24.00 | 0.72 (0.35-1.48) |
| Yes | 15 | 18.99 |  | 19 | 18.45 |  |
| Oil |  |  |  |  |  |  |
| No | 13 | 19.70 | 1.14 (0.60-2.15) | 1 | 8.33 | 3.05 (0.38-24.39) |
| Yes | 140 | 21.81 |  | 36 | 21.69 |  |
| Dairy |  |  |  |  |  |  |
| No | 144 | 21.98 | 0.73(0.35-1.52) | 37 | 22.70 | N.A. |
| Yes | 9 | 16.98 |  | 0 | 0.00 |  |
| Eggs |  |  |  |  |  |  |
| No | 153 | 21.76 | N.A. | 36 | 20.81 | 0.95(0.10-8.78) |
| Yes | 0 | 0.00 |  | 1 | 20.00 |  |
| 5 or more food groups |  |  |  |  |  |  |
| No | 131 | 22.13 | 1.17 (0.78-1.75) | 25 | 23.36 | 1.38 (0.74-2.57) |
| Yes | 22 | 18.97 |  | 12 | 16.90 |  |
| 4 or more food groups |  |  |  |  |  |  |
| No | 149 | 21.91 | 1.53 (0.61-3.84) | 35 | 21.60 | 1.73 (0.46-6.53) |
| Yes | 4 | 14.29 |  | 2 | 12.50 |  |
| **HOUSEHOLD VARIABLES** | | | | | | |
| **Sex head of household(HH)** |  |  |  |  |  |  |
| Male | 146 | 22.26 | 1.65 (0.82-3.34)* | 23 | 22.33 | 1.20 (0.66-2.17) |
| Female | 7 | 13.46 |  | 14 | 18.67 |  |
| **Age HH** |  |  |  |  |  |  |
| < 40 years | 65 | 18.36 | 0.75 (0.56-0.99)** | 24 | 21.62 | 1.28 (0.67-2.43) |
| ≥ 40 years | 86 | 24.64 |  | 11 | 16.92 |  |
| **HH literacy (read and write)** |  |  |  |  |  |  |
| Yes | 60 | 21.51 | 0.99 (0.74-1.31) | 25 | 25.77 | 1.74 (0.93-3.24)* |
| No | 93 | 21.83 |  | 12 | 14.81 |  |
| **Person in charge of food preparation (PCFP)** |  |  |  |  |  |  |
| Wife or HH(she) | 148 | 21.54 | 0.91 (0.41-1.97) | 33 | 22.92 | 1.95 (0.74-5.13) |
| Other | 5 | 23.81 |  | 4 | 11.76 |  |
| **Years of education of the PCFP** |  |  |  |  |  |  |
|  | 0,28 | 1.17 | 0.389 | 3,46 | 4.86 | 0.808 |
| **Number of people living in the house** |  |  |  |  |  |  |
|  | 6,45 | 1.68 | 0.716 | 5,14 | 1.72 | 0.663 |
| **Number of children in the house** |  |  |  |  |  |  |
|  | 3,06 | 1.14 | 0.007** | 2,19 | 0.94 | 0.880 |
| **DOES THE HOUSEHOLD….** |  |  |  |  |  |  |
| **own land?** |  |  |  |  |  |  |
| No | 4 | 23.53 | 1.09 (0.46-2.60) | 33 | 20.63 | 0.93 (0.37-2.32) |
| Yes | 149 | 21.56 |  | 4 | 22.22 |  |
| **have domestic animals or chicken?** |  |  |  |  |  |  |
| No | 6 | 23.08 | 1.07 (0.52-2.19) | 25 | 22.12 | 1.20 (0.65-2.22) |
| Yes | 147 | 21.55 |  | 12 | 18.46 |  |
| **cultivate teff?** |  |  |  |  |  |  |
| No | 39 | 16.96 | 1.53 (1.02-2.30)** | 37 | 21.26 | N.A. |
| Yes | 114 | 23.85 |  | 0 | 0.00 |  |
| **cultivate rice?** |  |  |  |  |  |  |
| No | 115 | 24.01 | 0.63 (0.42-0.95)** | 36 | 20.45 | 0.41 (0.10-1.69) |
| Yes | 38 | 16.59 |  | 1 | 50.00 |  |
| **cultivate millet?** |  |  |  |  |  |  |
| No | 140 | 21.91 | 1,16 (0.70-1.94) | 36 | 20.81 | 1.04 (0.18-6.15) |
| Yes | 13 | 18.84 |  | 1 | 20.00 |  |
| **cultivate beans?** |  |  |  |  |  |  |
| No | 148 | 21.57 | 0.95 (0.43-2,08) | 37 | 20.90 | N.A. |
| Yes | 5 | 22.73 |  | 0 | 0.00 |  |
| **consume products from their own cattle?** |  |  |  |  |  |  |
| Do not consume own cattle products | 12 | 18.75 | 1 | 34 | 20.73 | 1 |
| Consume own cattle products | 53 | 23.87 | 1.36 (0.68-2.74) | 0 | 0.00 | N.A. |
| Do not have cattle | 88 | 21.05 | 1.16 (0.59-2.26) | 2 | 18.18 | 0.85 (0.18-4.12) |
| **consume products from own goats?** |  |  |  |  |  |  |
| Do not consume own goat products | 140 | 22.08 | 1 | 36 | 21.05 | 1 |
| Consume own goat products | 5 | 14.29 | 0.59 (0.22-1.54) | 0 | 0.00 | N.A. |
| Do not have goat | 8 | 23.53 | 1.09 (0.48-2.45) | 1 | 14.29 | 0.63 (0.07-5.36) |
| **consume products from their own sheep?** |  |  |  |  |  |  |
| Do not consume own sheep products | 117 | 21.27 | 1 | 36 | 21.18 | 1 |
| Consume own sheep products | 15 | 24.19 | 1.18 (0.64-2.19) | 0 | 0.00 | N.A. |
| Do not have sheep | 20 | 22.47 | 1.07 (0.63-1.84) | 1 | 16.67 | 0.74 (0.08-6,57) |
| **consume products from their own chickens?** |  |  |  |  |  |  |
| Do not consume own chicken products | 70 | 24.48 | 1 | 27 | 20.93 | 1 |
| Consume own chicken products | 17 | 21.52 | 0.85 (0.46-1.54) | 0 | 0.00 | N.A. |
| Do not have chicken | 65 | 19.06 | 0.73 (0.50-1.06) | 10 | 21.28 | 1.02 (0.45-4.10) |
| *p<0.10; ** p<0.05 | | | | | | |
